# Supplementary material for: Long range correlations and slow time scales in a boundary driven granular model
Source: Sci Rep. 2021 Jul 9;11:14206. doi: 10.1038/s41598-021-93091-1 (PMC8270907; doi:10.1038/s41598-021-93091-1)
Supplement: Supplementary file 1 — Supplementary Information 1. [file 41598_2021_93091_MOESM1_ESM.pdf]

# Long range correlations and slow time scales in a boundary driven granular model: Supplemental Materials

Andrea Plati:
Dipartimento di Fisica, Università di Roma Sapienza, P.le Aldo Moro 2, 00185 Roma, Italy
Andrea Puglisi:
Istituto dei Sistemi Complessi - CNR and Dipartimento di Fisica, Università di Roma Sapienza, P.le Aldo Moro 2, 00185, Rome, Italy
INFN, University of Rome Tor Vergata, Via della Ricerca Scientifica 1, 00133 Rome, Italy

## S1: Subleading terms in the large system limit

Here we show how performing the large system limit ($L \gg 1$) subleading terms $\sim 1/L$ occur. Starting from Eq.10 of the main text we consider the contribution proportional to $b_L^2$:

$$b_L^2 \Pi^2 \sum_{lk} \frac{\sin(jl\Pi)\sin(mk\Pi)\sin(lL\Pi)\sin(kL\Pi)}{\Delta(\alpha) - \cos(k\Pi) - \cos(l\Pi)} \tag{1}$$

where $\Pi = \pi/(L+1)$ and we note that: $\sin(lL\Pi)\sin(kL\Pi) = (-1)^{k+l+2}\sin(l\Pi)\sin(k\Pi)$. Considering a generic function $f$ we can write

$$\Pi^2 \sum_{lk} (-1)^{k+l+2} f(jl\Pi, mk\Pi) = \Pi^2 \sum_{nh} \big[ f(2jn\Pi, 2mh\Pi) - f(2jn\Pi + j\Pi, 2mh\Pi) + f(2jn\Pi + j\Pi, 2mh\Pi + m\Pi) - f(2jn\Pi, 2mh\Pi + m\Pi) \big] \tag{2}$$

that taking the large system limit $L \gg 1$ and replacing sums with integrals as $\Pi \sum_{m=0}^{m=L/2} f(2m\Pi) \to \frac{1}{4}\int_0^\pi dx f(x)$ becomes:

$$\frac{1}{4}\int_0^\pi dz ds \left[ f(jz, ms) - f(jz + j\Pi, ms) + f(jz + j\Pi, ms + m\Pi) - f(jz, ms + m\Pi) \right] \sim \mathscr{O}(1/L), \quad L \gg 1, \quad m \vee j \ll L \tag{3}$$

because all the terms at the zeroth order vanish in the integrand. This explains why it is possible to neglect the term proportional to $b_L^2$ in Eq. 10 of the main text once the large system limit is taken and for $j \vee m$ small enough. This is consistent with the idea that the effect of the bath acting on the $L$th site can be neglected only if $\sigma_{jm}$ is calculated for sites that are far away from $L$.

## S2: Covariance matrix in the NHHP

Here we give some details about the calculations necessary to derive the asymptotic predictions of Eqs. 14 from Eq. 13 of the main text. To do so we start from the latter equation in a form more suitable for next calculations:

$$\sigma_{jm}^{\text{NHHP}} = \lim_{L\to\infty} \frac{4T_1}{\pi^2} \int_{\frac{\pi}{L+1}}^{\frac{\pi L}{L+1}} dz \int_{\frac{\pi}{L+1}}^{\frac{\pi L}{L+1}} ds \sin(jz)\sin(ms) g(z,s) \quad \text{where} \quad g(z,s) = \frac{\sin(z)\sin(s)}{2 - \cos(z) - \cos(s)}. \tag{4}$$

In this expression we have shown the explicit form of the large $L$ limit because the integrand of the function $g$ is a function of both $z$ and $s$ that is singular in the point $(0,0)$. Indeed, its right value in the origin comes from the limit for large $L$ of the integration domain $[\frac{\pi}{L+1}, \frac{\pi L}{L+1}] \times [\frac{\pi}{L+1}, \frac{\pi L}{L+1}]$ in the $zs$ plane. More specifically we have that $0 \le g(z,s) \le 1 \; \forall z,s \in [0,\pi]$ and that $\lim_{z\to 0} g(z^a, z^b) \sim z^{a-b}$ if $a \ge b$. In the remainder, we consider the integration intervals as $[\frac{\pi}{L+1}, \pi]$ because the singularity is just in the origin. Integrating two times by parts and noting that $g(\pi, s) = g(z,\pi) = 0 \; \forall \; z,s$ we have:

$$\sigma_{jm}^{\text{NHHP}} = \lim_{L\to\infty} \frac{4T_1}{\pi^2 jm} \left[ \cos\left(\frac{j\pi}{L+1}\right)\cos\left(\frac{m\pi}{L+1}\right) g\left(\frac{\pi}{L+1}, \frac{\pi}{L+1}\right) + \cos\left(\frac{m\pi}{L+1}\right) \int_{\frac{\pi}{L+1}}^{\pi} dz \cos(jz)\, \partial_z g\left(z, \frac{\pi}{L+1}\right) + \cos\left(\frac{j\pi}{L+1}\right) \int_{\frac{\pi}{L+1}}^{\pi} ds \cos(ms)\, \partial_s g\left(\frac{\pi}{L+1}, s\right) + \int_{\frac{\pi}{L+1}}^{\pi} ds dz \cos(jz)\cos(ms)\, \partial_{zs} g(z,s) \right]. \tag{5}$$

We want to show that $\sigma_{jm}^{\text{NHHP}} \sim (jm)^{-1}$ so we have to demonstrate that the sum of the terms in the square brackets is $\mathscr{O}(1)$ for $m, j \gg 1$ in the large $L$ limit. The first term clearly tends to 1 when $L \to \infty$ regardless the value of $j$ and $m$ (remember that $j, m \ll L$). Reintroducing $\Pi = \pi/(L+1)$ we can express Eq. (5) as:

$$\sigma_{jm}^{\text{NHHP}} \sim \frac{4T_1}{\pi^2 jm} [1 + C_{jm}] \quad \text{where} \quad C_{jm} = \lim_{L\to\infty} [\cos(m\Pi) I_j + \cos(j\Pi) I_m + I_{jm}] \tag{6}$$

and where $I_j$, $I_m$ and $I_{jm}$ are respectively the integrals of the second, third and fourth term in the square brackets of Eq. (5). The estimate of the asymptotic behavior of such integrals is not trivial because of the presence of the derivatives of $g(z,s)$ that diverge in the origin. We then proceed by estimating upper bounds. It is important to note that, in order to demonstrate $\sigma_{jm}^{\mathrm{NHHP}} \sim (jm)^{-1}$, requiring $C_{jm} \sim \mathscr{O}(1)$ or $|C_{jm}| \leq 1$ is not enough because it would bring contributions as $-1 \pm o(1/j)$ that imply the emergence of a faster decay. The right thing to do is instead to show that $|C_{jm}| \leq c$ with $c < 1$. In this way, we could be sure that $C_{jm}$ cannot cancel 1 in Eq. (6). Starting by $I_j$, we define $u(z) = \partial_z g(z, \frac{\pi}{L+1})$ and rewrite it as:

$$I_j = \int_{\frac{\pi}{L+1}}^{\pi+\frac{\pi}{L+1}} dz \cos(jz)\, u(z) + \mathscr{O}(1/L) \tag{7}$$

Now we note that the interval of integration is much larger than the period $T_j = \frac{2\pi}{j}$ of the cosine so we can split it in a sum of contributions over consecutive periods. Without loss of generality we can assume $j$ even and exploit the periodicity of the cosine obtaining:

$$I_j = \sum_{k=1}^{k=j/2} \int_{(k-1)T_j+\Pi}^{kT_j+\Pi} dz \cos(jz) u(z) = \frac{1}{j} \int_{\Pi}^{2\pi+\Pi} dx \cos(x) \sum_{k=1}^{k=j/2} u\left(\frac{x}{j} + (k-1)T_j\right) \tag{8}$$

where we have have changed variable as $x = jz + 2\pi(k-1)$ and reintroduced the symbol $\Pi = \frac{\pi}{L+1}$. Now we use the fact that $T_j \ll 1$ to exchange the sum over $k$ with an integral as $\sum_k f((k-1)T_j) \to T_j^{-1} \int d\phi_j f(\phi_j)$ and return to an expression with $g$:

$$I_j = \frac{1}{2\pi} \int_{\Pi}^{2\pi+\Pi} dx \cos(x) \int_0^{\pi-\frac{2\pi}{j}} d\phi_j u\left(\frac{x}{j} + \phi_j\right) = \frac{1}{2\pi} \int_{\Pi}^{2\pi+\Pi} dx \cos(x) \left[g\left(\frac{x}{j} + \pi - \frac{2\pi}{j}, \Pi\right) - g\left(\frac{x}{j}, \Pi\right)\right]. \tag{9}$$

The function $g$ can be regularly expanded in series around the point $(\pi, 0)$. Doing this, it's easy to verify that the integral of the first term in the brackets gives $\mathscr{O}(1/j)$ contributions. We can't perform such an estimate for $g(x/j, \Pi)$ because the derivatives near the origin are not well defined. Nevertheless, we know that $g(x/j, \Pi) \in [0,1]\ \forall\, x \in [\Pi, 2\pi/+\Pi]$ if $j$ is sufficiently large so we can estimate an upper bound for $I_j$ (and $I_m$) as: $\lim_{L\to\infty} |I_{j(m)}| \leq 1/\pi$ for $j \gg 1$. This happens because, given $T$ a $2\pi$-large interval with $T_{+(-)}$ the sub-interval where the cosine is positive(negative) and $g(x) \in [0,1]$ if $x \in T$, we can write:

$$\left|\int_T \cos(x) g(x)\right| = \left|\left|\int_{T_+} \cos(x) g(x)\right| - \left|\int_{T_-} \cos(x) g(x)\right|\right| \leq \frac{1}{2} \int_T |\cos(x)| = 2 \tag{10}$$

With the same kind of calculations leading to Eq. (9) we obtain:

$$I_{jm} = \frac{1}{4\pi^2} \int_{\Pi}^{2\pi+\Pi} dx dy \cos(x) \cos(y) g\left(\frac{x}{j}, \frac{y}{m}\right) + \mathscr{O}((mj)^{-1}). \tag{11}$$

Using inequalities similar to the ones of Eq. (10) but for 2D integrals we estimate the upper bound of Eq. (11) as $\lim_{L\to\infty} |I_{jm}| \leq 2/\pi^2$ for $j,m \gg 1$. Putting together these results in the definition of $C_{jm}$ of Eq. (6) we are sure that in the large $L$ limit:

$$|C_{jm}| \leq \lim_{L\to\infty} [|I_j| + |I_m| + |I_{jm}|] = \frac{2}{\pi}\left(1 + \frac{1}{\pi}\right) \simeq 0.83926 < 1 \quad \text{for} \quad j,m \gg 1 \tag{12}$$

We conclude that $\sigma_{jm}^{\mathrm{NHHP}} \sim (jm)^{-1}$ from which Eq. 14a of the main text is straightforward.

It is important to note that, in order to obtain Eqs. (8) and (9), we need both $j$ and $m \gg 1$. So we have to use another way to estimate the asymptotic behavior of $\sigma_{1m}^{\mathrm{NHHP}}$. It can be rewritten as

$$\sigma_{1m}^{\mathrm{NHHP}} = \frac{4T_1}{\pi^2} \int_0^{\pi} dz ds \sin(ms) g_1(s,z) \quad \text{where} \quad g_1(s,z) = \frac{\sin^2(z)\sin(s)}{2 - \cos(z) - \cos(s)} \tag{13}$$

and $g_1$ is regular in the origin because $\lim_{z\to 0} g_1(z^a, z^b) = 0\ \forall\ a,b > 0$. We can perform the integral over $z$ obtaining $\int_0^{\pi} dz g_1(z,s) = \pi\left[-2 + \cos(s) + \sqrt{6 - 2\cos(s)}\sin(s/2)\right]\sin(s)$ where the first two terms in the brackets vanish when also the integral over $s$ is performed ($m$ is an integer). We have now that $\sigma_{1m}^{\mathrm{NHHP}} = \frac{4T_1}{\pi^2} \int_0^{\pi} ds \sin(ms) f(s)$ where $f(s) = \sin(s)\left[\sqrt{6 - 2\cos(s)}\sin(s/2)\right]$. Integrating four times by parts and noting that $f(0) = f(\pi) = f''(\pi) = 0$ while $f''(0) = 2$ we obtain:

$$\sigma_{1m}^{\mathrm{NHHP}} = \frac{8T_1}{\pi m^3} + R_m \sim \frac{8T_1}{\pi m^3} + \mathscr{O}(m^{-5}), \quad m \gg 1 \tag{14}$$

where $R_m = (m)^{-4}(\pi)^{-1}\int_0^\pi ds \sin(ms) f^{(4)}(s)$ so $|R_m| \le (m)^{-4}(\pi)^{-1}|\max(f^{(4)}(s))| \int_0^\pi ds |\sin(ms)| = 2(m)^{-5}(\pi)^{-1}|\max(f^{(4)}(s))| \simeq 19(m)^{-5}(\pi)^{-1}$ . The last quantity needed for the Eqs. 14 of the main text is $\sigma_{11}^{\mathrm{NHHP}} = \int_0^\pi dz ds \sin(z)\sin(s) g(z,s) = \pi^2 - 8\pi/3$ that is finite and does not depend on $m$ so the asymptotic behavior for $\zeta_{1m}$ directly follows from the ones derived for Eqs. 14a and 14b of the main text.

## S3: Covariance matrix in the HHP

In order to derive Eq. 16 from Eq. 10 of the main text we have to discuss the contributions coming from the sum $\sum_n b_n^2 \sin(ln\Pi)\sin(kn\Pi)$ that compares in the latter. As explained in the first appendix, the term proportional to $b_L^2$ gives a subleading term $\mathscr{O}(1/L)$ in the large system limit while the one proportional to $b_1^2$ gives $4T_1(1+\alpha)(\pi)^{-2}\Sigma_{jm}(\alpha)$. Regarding the other contributions, we exploit orthogonality to express the remaining sum as:

$$\sum_{n=2}^{n=L-1} \sin(ln\Pi)\sin(kn\Pi) = \frac{L+1}{2}\delta_{kl} - \sin(l\Pi)\sin(k\Pi) - \sin(lL\Pi)\sin(kL\Pi) \tag{15}$$

where again the last term gives $\mathscr{O}(1/L)$ for $L \gg 1$. Thus, using this equation and neglecting subleading terms, Eq. 10 of the main manuscript becomes:

$$\sigma_{jm}(\alpha) = \Pi^2 \sum_{lk} \frac{\sin(jl\Pi)\sin(mk\Pi)}{\Delta(\alpha) - \cos(k\Pi) - \cos(l\Pi)} \left[\frac{2\alpha T_a(L+1)}{\pi^2}\delta_{kl} + \frac{4T_1}{\pi^2}\left(1+\alpha\left(1-\frac{T_a}{T_1}\right)\right)\sin(l\Pi)\sin(k\Pi)\right] \tag{16}$$

that in the large system limit gives Eq. 16 of the main manuscritp.

In the main text we proceed from Eq. 16 by considering constant amplitude of noises i.e. $T_1 = T_a\gamma_a/(\gamma+\gamma_a)$. In this way the term proportional to $\Sigma(\alpha)$ vanishes and one can shorten calculations concentrating just on the integral over $z$. To verify that the asymptotic behavior of Eq. 19 of the main text holds also without constant amplitude of noises we have to show that $\Sigma_{jm}(\alpha)$ does not decay slower than $\exp(-\sqrt{\alpha}m)$. We then consider the fourier transform $\tilde{\Sigma}_{j\omega}(\alpha) = \int dm \exp(i\omega m)\Sigma_{jm}(\alpha)$ for small $\omega$:

$$\tilde{\Sigma}_{j\omega} \sim \int_0^\pi dz \frac{\sin(jz)\sin(z)\omega}{1+\alpha-\cos(z)+\frac{\omega^2}{2}} \quad \text{so} \quad \Sigma_{jm} \sim \int_0^\pi dz \frac{\sin(jz)\sin(z)}{1+\alpha-\cos(z)} \exp(-m\sqrt{2(1+\alpha-\cos(z))}) \tag{17}$$

and for this last expression is simple to show that $|\Sigma_{jm}| \le \frac{\pi}{\alpha}\exp(-\sqrt{2\alpha}m)$. Then we are sure that its behavior for large $m$ will be subleading with respect to $\exp(-\sqrt{\alpha}m)$.

To complete the discussion about the exponential decay in the HHP we need to evaluate the result of Eq. 18 of the main manuscript. We then write such integral after one integration by parts obtaining:

$$\frac{2\alpha T_a}{\pi}\int_0^\pi dz \frac{\sin^2(mz)}{\Delta(\alpha)-2\cos(z)} = \frac{2\alpha T_a}{\pi}\left[\frac{\pi}{2(4+\alpha)} - \int_0^\pi dz \frac{z\sin(z)}{(\Delta(\alpha)-2\cos(z))^2} - \int_0^\pi dz \frac{\sin(mz)\sin(z)}{2m(\Delta(\alpha)-2\cos(z))^2}\right] \tag{18}$$

from which we have that $\sigma_{mm}^{\mathrm{HHP}}(\alpha) = T_a\sqrt{\frac{\alpha}{4+\alpha}} + o(m^{-1})$.

## S4: Spatial correlation in the cooling state

An important question that often arises in granular systems regards the relation between the properties of the cooling dynamics and the one of the NESS obtained with the injection of energy. In our case we obtain the cooling state by switching off all the temperatures in the lattice (matrix $\hat{B}$ with all zero entries). In this situation the covariance matrix is simply given by Eq. 7a of the main text. Where the brackets $\langle\rangle$ refer to a mean on the initial conditions. Exploiting the symmetry of $\hat{A}$ we can rewrite it as:

$$\sigma_{jm}(t,s) = \sum_{nhkl} S_{hn} e^{-\lambda_n t} S^+_{nk} \langle v_k(0) v_l(0)\rangle S_{lh} e^{-\lambda_h s} S^+_{hj} \tag{19}$$

Keeping initial conditions identically and independently distributed around 0 with the variance 1 so that $\langle v_k(0)v_l(0)\rangle = \delta_{kl}$ and exploiting orthogonality of the eigenvectors we have:

$$\sigma_{jm}(t,s) = \sum_n S_{jn} e^{-\lambda_n(t+s)} S^+_{nm} \tag{20}$$

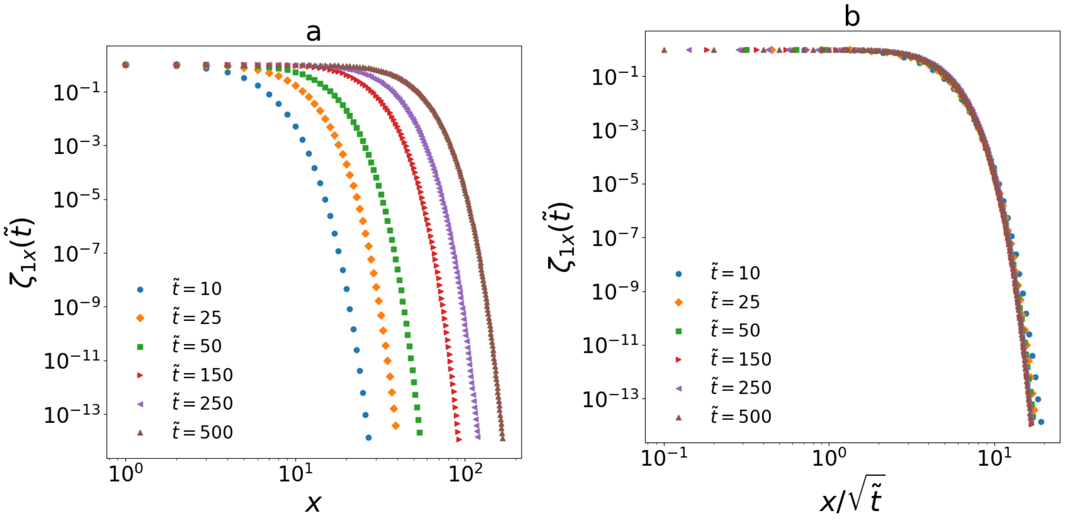


**Figure 1.** Spatial correlation function in the cooling state after different times $\tilde{t}$ (a). We observe a collapse by rescaling the horizontal axis by $\sqrt{\tilde{t}}$ (b). Here we have considered $L = 200$.

That in the Toeplitz case for $t = s$ becomes:

$$\sigma_{jm}(t) = \frac{\exp(-2(2\gamma+\gamma_a)t)\Pi}{\pi} \sum_n \sin(jn\Pi)\sin(nm\Pi)\exp(4\gamma t\cos(n\Pi)) . \tag{21}$$

where we note that for $t = 0$ $\sigma_{jm}(0) = \delta_{jm}$ as imposed by the initial state. The same uncorrelated condition, expected for non-iteracting systems, is also obtained with $\gamma = 0$. Another important properties of the $\sigma_{ij}(t)$ is that the dependence on $\gamma_a$ is factored out from the sum so, when calculating $\zeta_{jm} = \sigma_{jm}/\sqrt{\sigma_{jj}\sigma_{mm}}$, it simplifies. Moreover, also the dependence from $\gamma$ can be removed just by using the adimensional time $\tilde{t} = \gamma t$. To conclude, during the cooling the behavior of spatial correlations is crucially different from the one observed in the two heated phases studied in the main text. In particular, the parameter $\alpha$ does not play a crucial role as in the NESS. This is an intriguing result because we found that an external source of energy makes something more than just keeping alive the dynamics that characterizes the system when it cools down.

In Fig. 1 we show $\zeta_{1x}(\tilde{t})$ for different times $\tilde{t}$ and we clearly observe that it presents a finite cutoff that grows with the delay time $\tilde{t}$. We can understand it by thinking that the information is propagating through the system in time. In Fig. 1b we show how rescaling the space with $\sqrt{\tilde{t}}$ all the curves collapse. So the information propagates as $\xi(t) \propto \sqrt{\gamma t}$. This result is fully consistent with diffusion-like coarsening dynamics of vortices, found in other models for granular velocity fields[1–3]. In those models however the cooling state is closer to "dilute" situations where interactions are sequences of separate binary collisions.

## S5: Reintroduction of space and connection with active matter

Although it is reasonably justified from empirical observations, neglecting the positional dynamics remains the main approximation of our model. A way to reintroduce it in our description is to consider a harmonic potential between nearest neighbors in the lattice. The equation of motion for each particle would then be of this form

$$\dot{x}_i = v_i \tag{22a}$$

$$\dot{v}_i = -(\gamma_{a(b)} + 2\gamma)v_i - 2kx_i + k(x_{i+1} + x_{i-1}) + \gamma(v_{i+1} + v_{i-1}) + \sqrt{2T_{a(i)}\gamma_{a(b)}}\xi_i(t) \tag{22b}$$

where we consider again a bath on the boundaries characterized by ($\gamma_b$, $T_{1(L)}$) and a bath on the bulk ($\gamma_a$, $T_a$).

It is interesting to note that we can obtain equations of the same form when considering a 1D chain of (overdamped) active particles with harmonic interactions, where self-propulsion is modeled using a colored noise $\eta$ (Active Ornstein-Uhlenbeck Particles AOUP):

$$\dot{x}_i = -k(x_i - x_{i+1}) - k(x_i - x_{i-1}) + \eta_i(t) \tag{23a}$$

$$\dot{\eta}_i = -\gamma_a\eta_i + \sqrt{2T_a\gamma_a}\xi_i(t) \tag{23b}$$

where $\xi_i$ are Gaussian white noises with unitary variance. Time-deriving the first of these equations and following standard manipulations, we get[4]:

$$\dot{x}_i = v_i \tag{24a}$$

$$\dot{v}_i = -(\gamma_a + 2k)v_i - 2k\gamma_a x_i + k\gamma_a(x_{i+1} + x_{i-1}) + k(v_{i+1} + v_{i-1}) + \sqrt{2T_a\gamma_a}\xi_i(t) \tag{24b}$$

which are formally equivalent to Eqs. (22). If we consider the particles fixed on the lattice and neglect the positional dynamics we find the analogous of the granular case studied in the main with a transition in $\gamma_a = 0$. While in the granular chain removing the bath on the bulk has a specific and realistic physical condition (granular materials are often driven only through boundaries) in the active case it seems meaningless. A self-propelled harmonic chain modeled by Eqs. (24) has been studied taking account the positional dynamics and assuming spatially homogeneous self-propulsion[5]. The authors perform calculations based on translational invariance (they solve the system in the Bravais reciprocal lattice). This assumption is crucial and it is also the main difference with our approach in which we are interested in the effect of non-homogeneous heating. The interesting connection with our investigation is that they found a correlation length that scales as $\xi \sim \sqrt{1/\gamma_a}$ as in our case[5].

The study of correlations in this kind of 1D systems with both positional dynamics and non-homogeneous heating is, up to our knowledge, still lacking. We are currently working in this direction.

## References


**1.** Van Noije, T., Ernst, M., Brito, R. & Orza, J. Mesoscopic theory of granular fluids. *Phys. Rev. Lett.* **79**, 411 (1997).

**2.** Baldassarri, A., Marini Bettolo Marconi, U. & Puglisi, A. Cooling of a lattice granular fluid as an ordering process. *Phys. Rev. E* **65**, 051301, DOI: 10.1103/PhysRevE.65.051301 (2002).

**3.** Baldassarri, A., Puglisi, A. & Sarracino, A. Coarsening in granular systems. *C. R. Physique* **16**, 291–302 (2015).

**4.** Maggi, C., Marconi, U. M. B., Gnan, N. & Di Leonardo, R. Multidimensional stationary probability distribution for interacting active particles. *Sci. reports* **5**, 10742 (2015).

**5.** Caprini, L. & Marconi, U. M. B. Time-dependent properties of interacting active matter: Dynamical behavior of one-dimensional systems of self-propelled particles. *Phys. Rev. Res.* **2**, 033518, DOI: 10.1103/PhysRevResearch.2.033518 (2020).
